# Nebulization of risedronate alleviates airway obstruction and inflammation of chronic obstructive pulmonary diseases via suppressing prenylation-dependent RAS/ERK/NF-κB and RhoA/ROCK1/MLCP signaling

**DOI:** 10.1186/s12931-022-02274-5

**Published:** 2022-12-28

**Authors:** Di Liu, Wen Xu, Yuan Tang, Jingxue Cao, Ran Chen, Dingwei Wu, Hongpeng Chen, Bo Su, Jinfu Xu

**Affiliations:** 1 Central Laboratory, Shanghai Pulmonary Hospital, School of Medicine, Tongji University, Shanghai, People’s Republic of China; 2 Department of Radiation Oncology, Shanghai Pulmonary Hospital, School of Medicine, Tongji University, Shanghai, People’s Republic of China; 3 Department of Respiratory and Critical Care Medicine, Shanghai Pulmonary Hospital, School of Medicine, Tongji University, Shanghai, People’s Republic of China; 4 Basic Medical College, Bengbu Medical College, Bengbu, People’s Republic of China; 5 Department of Radiology, Shanghai Pulmonary Hospital, School of Medicine, Tongji University, Shanghai, People’s Republic of China; 6 Zhejiang Xianju Pharmaceutical Co., Ltd, Xianju, People’s Republic of China; 7 School of Life Sciences, Bengbu Medical College, Bengbu, People’s Republic of China

**Keywords:** Pulmonary disease, Chronic obstructive, Risedronic acid, Inflammation, Animal models

## Abstract

**Background:**

Chronic obstructive pulmonary disease (COPD) is a progressive disorder that causes airway obstruction and lung inflammation. The first-line treatment of COPD is the bronchodilators of β2-agonists and antimuscarinic drugs, which can help control the airway obstruction, but the long-term use might render the drug tolerance. Bisphosphonates are widely used in osteoclast-mediated bone diseases treatment for decades. For drug repurposing, can delivery of a third generation of nitrogen-containing bisphosphonate, risedronate (RIS) ameliorate the progression of COPD?

**Methods:**

COPD rats or mice models have been established through cigarette-smoking and elastase injection, and then the animals are received RIS treatment via nebulization. Lung deposition of RIS was primarily assessed by high-performance liquid chromatography (HPLC). The respiratory parameters of airway obstruction in COPD rats and mice were documented using plethysmography method and resistance-compliance system.

**Results:**

High lung deposition and bioavailability of RIS was monitored with 88.8% of RIS input dose. We found that RIS could rescue the lung function decline of airspace enlargement and mean linear intercept in the COPD lung. RIS could curb the airway obstruction by suppressing 60% of the respiratory resistance and elevating the airway’s dynamic compliance, tidal volume and mid-expiratory flow. As an inhibitor of farnesyl diphosphate synthase (FDPS), RIS suppresses FDPS-mediated RAS and RhoA prenylation to obstruct its membrane localization in airway smooth muscle cells (ASMCs), leading to the inhibition of downstream ERK-MLCK and ROCK1-MLCP pathway to cause ASMCs relaxation. Additionally, RIS nebulization impeded pro-inflammatory cell accumulation, particularly macrophages infiltration in alveolar parenchyma. The NF-κB, tumor necrosis factor-alpha, IL-1β, IL-8, and IL-6 declined in microphages following RIS nebulization. Surprisingly, nebulization of RIS could overcome the tolerance of β2-agonists in COPD-rats by increasing the expression of β2 receptors.

**Conclusions:**

Nebulization of RIS could alleviate airway obstruction and lung inflammation in COPD, providing a novel strategy for treating COPD patients, even those with β2-agonists tolerance.

**Supplementary Information:**

The online version contains supplementary material available at 10.1186/s12931-022-02274-5.

## Introduction

Chronic obstructive pulmonary disease (COPD) is a public health issues associated with economic and social burdens [1], with approximately three million deaths annually and 8.6% of adult new cases [2–4]. Airway obstruction, as the principal feature of COPD, is progressive and is caused by mucus hypersecretion, small airway loss, airway remodeling, and lung inflammation [5]. Airway remodeling, including epithelial hyperplasia and metaplasia, increased airway wall thickness, and dysfunction of airway smooth muscle (ASM), results in emphysema of COPD [6]. The smooth muscle cells are pivotal components of the airway, mediated the generation of protease, inflammatory factors, and growth factors. The dysfunction of ASM, including contractile function alteration and the increase of ASM, renders the airway inflammation and remodeling in COPD [7].

The bronchodilator medications, such as beta-2(β2) agonists (e.g., albuterol, salmeterol, and formoterol), and anticholinergic agents (e.g., ipratropium) are widely used to relax ASM to alleviate the shortness of breath by stimulating G protein-cAMP/PKA signaling pathway and blocking the muscarinic receptors in ASM, respectively [8, 9]. Nevertheless, these drugs cannot renovate COPD’s airway remodeling [7], and the chronic use might unfortunately lead to development of tolerance. The downregulation of beta-2 adrenergic receptor (β2-AR) contributes to the β2 agonists’ resistance in COPD patients [10]. Finding a new strategy that could alleviate ASM dysfunction and reverse the airway remodeling is important. The previous study screened one novel regulator of β2-AR through shRNA library, farnesyl diphosphate synthase (FDPS, also known as farnesyl pyrophosphate synthase, FPPS), associating with β2-AR downregulation [11].

As an inhibitor of FDPS, nitrogen-containing bisphosphonates, alendronate could inhibit the downregulation of β2-AR to overcome the tolerance of β2 agonists [11]. Kurabayashi et al*.* further validated that alendronate could attenuate the emphysema in COPD mice [12]. However, the influence of alendronate on ASM was not detected in COPD mice. And the concentration of alendronate used to mice was high, limiting the clinical transition of alendronate on COPD patients. Herein, we aim to explore the influence of another FDPS inhibitor, risedronate (RIS), the latest generation of diphosphonates [13], and an FDA-approved drug for osteoporosis treatment, on airway of COPD rodent models.

The COPD rats and mice models were constructed to explore the drug response of RIS and its molecular mechanisms. FDPS, a critical regulator of mevalonate pathway, could catalyze post-translational prenylation, which is pivotal for the membrane binding of GTPases. Hence, we focused on the GTPases of KRAS (kirsten rat sarcoma virus) and RhoA (ras homolog family member A) signaling to explore the molecular mechanism of RIS. The change of pulmonary morphology and pulmonary function was used to assess the effect of RIS on COPD rats and mice. The β2 agonists resistant COPD rats were also established to investigate the response of RIS.

## Methods

### Animals and treatment

Female four-six-week-old Sprague Dawley rats (n = 50) weighting 85-160 g ± 5 g and four- week-old Balb/c mice (n = 15) weighting 15 ± 2 g were purchased from shanghai Slac Laboratory Animal Co. Ltd (SPF grade, Experiment Animal Co., Ltd., Shanghai, China). The animals were raised and maintained at a pathogen-free sterile barrier facility (temperature, 22 ◦C; humidity, 50%-60%) under a 12-h light/dark cycle at Shanghai Pulmonary Hospital. All the animals were provided with standard rodent diet and water ad libitum. An acclimatization period of seven days was allowed before any initiation in experimental work.

The rats and mice were subjected to the passive smoking of five unfiltered cigarettes for three times daily (15–20 min for each time), 7 days a week for up to 18 weeks and 12 weeks, respectively. An automated cigarette-smoking apparatus (Model TE-10, Teague Enterprises, Davis, CA) was used for cigarette exposure as previously described [14]. The control groups were exposed to fresh air. We simultaneously instilled 200 μl of LPS (lipopolysaccharide, 0.5 mg/L) into the tracheal of mice twice a week for up to four weeks to promote the development of COPD via inducing chronic inflammation. All animals were sequentially administered with 4.8 units/100 g body weight of porcine pancreatic elastase (Sigma-Aldrich Corp. St. Louis, USA) via instillation at week 17 and week 7 in rats and mice, respectively. The control groups were administered with saline. A few rats (n = 5) were sacrificed at week 12 for testifying the change of the pulmonary morphology and function. A few COPD rats (n = 20) were continuous nebulized salbutamol for three weeks to construct salbutamol-resistant COPD rats. We nebulized the RIS (20 µg) in salbutamol-resistant COPD rats for two weeks for exploring the long-term effect of RIS in vivo. All animals were euthanasia with carbon dioxide (CO2) at the end of point.

The RIS (HY-B0148, MCE, New Jersey, USA) was dissolved in PBS and salbutamol (HY-B1037, MCE, New Jersey, USA) was dissolved in ethanol, diluted in PBS with 1:1000 before using. The rats and mice were randomly divided into six groups. The useful data of respiratory parameters were measured from at least four different animals in each group. Group I is the normal rats and mice that measured respiratory parameters. Group II is control group of COPD rats and mice treated with PBS. Group III are the COPD rats and mice administered with RIS nebulization at different concentrations (0, 0.31, 0.625, 1.25, 2.5, and 5 mg/ml). Group IV is the COPD rats received salbutamol (0.125, 0.25, 0.5, 1 and 2 mg/ml) treatment. Group V are the salbutamol-resistant COPD rats treated with salbutamol nebulization at different concentrations (0.125, 0.25, 0.5, 1 and 2 mg/ml). Group VI are the salbutamol-resistant COPD rats treated with different concentration of RIS (0, 0.31, 0.625, 1.25, 2.5, and 5 mg/ml).

### Respiratory parameters

Non- invasive and invasive mechanics were used to monitor a few respiratory parameters representing lung functional ventilatory and bronchoconstriction of rats and mice. For rats, the lung function parameters mainly include specific airway resistance (sRAW), tidal volume (TV), peak expiratory flow (PEF), peak inspiratory flow (PIF), and mid-expiratory flow (EF50). For mice, the respiratory parameters mainly include TV, PEF, PIF, minute ventilation volume (MV), airway resistance (RI) and dynamic compliance (Cdyn). The different methods were utilized to assess these parameters in order to obtain more comprehensive results of RIS efficiency on COPD animals.

#### Non-invasive airway mechanics using the plethysmography method

The ventilatory and bronchoconstriction parameters, sRAW, TV, PEF, PIF, EF50 and MV of rats or mice were directly recorded with a software program of Buxco® Non-Invasive Airway Mechanics (NAM) (Data Sciences International [DSI], St. Paul, MN, USA) using the Plethysmography method without anesthesia. The rats or mice were restrained in a chambered special box which allows the independent measurement of nasal, mouth, thoracic and abdomen flows. The signal difference could be calculated to determine the specific airway resistance. The patented feature of NAM could detect the pulmonary function of animals under normal breath (Additional file 1: Fig. S1).

#### Invasive resistance and compliance system

The respiratory parameters, RI and Cdyn of mice were directly monitored by Buxco® FinePointe Resistance and Compliance system (RC) (Data Sciences International [DSI], St. Paul, MN, USA). The mice were anesthetized and instrumented with a detector in the trachea to directly measure RI and Cdyn after RIS treatment.

The above systems both have a small nebulizer that creates the mist out of the liquid agents. All the respiratory parameters of the animals were measured 5 min after the treatment of saline, RIS or salbutamol. The baseline of the parameters of animals was monitored before the treatment. The differences between the treated parameters and baseline parameters were documented as %change. Subsequently, the animals were sacrificed to collect bronchoalveolar lavage fluids (BALFs), and lung tissue samples for further experiments.

### Cell culture

#### The primary culture of rats’ airway smooth muscle cells

Primary culture of airway smooth muscle cells (ASMCs) of rats were prepared based on the differential adhesion of different cells [15, 16]. Briefly, the rats were anesthetized, and the trachea smooth muscle was separated. The isolated airway smooth muscle tissue was cut into a 1mm^3^ pieces and then transferred to a 10 cm cell culture dish for 30 min. The primary cells were cultured with 10 ml of Ham's F-12 medium (Thermo Fisher Scientific, South San Francisco, CA) containing 10% fetal bovine serum (FBS) (Thermo Fisher Scientific, South San Francisco, CA) in a 37 °C incubator. The cells obtained from airway smooth muscle might mix tracheal epithelial cells and fibroblasts besides airway smooth muscle cells (ASMCs). The tracheal epithelial cells and fibroblasts adhered to the cell dish within half an hour, while ASMCs generally required at least 1–4 h. Because of the different attachment times, ASMCs can be purified in the culture dish. When the cells achieved a confluence of 80%, the cells detached with 0.25% trypsinization to prepare a cell suspension which was then transferred into a new dish. After 1-h of cell culture, the upper cell suspension containing unattached ASMCs was aspirated to a new dish. ASMCs could be purred by repeating this process for four-five times.

The NR8383 alveolar macrophage cells were purchased from ATCC and cultured with Ham's F12 medium with 2 mM L-glutamine (Thermo Fisher Scientific, South San Francisco, CA) and 15% FBS under 37 °C and 5% CO2. The NR8383 cells received the treatment of 100 µm RIS for 48 h. The ASMCs received different concentrations RIS treatment for 24 h. The inflammation response was induced by LPS (100 ng/ml) (MCE, New Jersey, USA) stimulation for 30 min.

#### Inflammatory cell count in bronchoalveolar lavage fluids (BALFs)

BALFs collected from the pulmonary tissues as previously described [17]. The lavage fluids were centrifuged for 15 min at 1600 × g. The supernatant was stored at − 80 °C, and the pelleted cells were resuspended in PBS for inflammatory cells analysis. 1 × 10^5^ cells were subjected to cytospin centrifugation on glass slides, and fixed with 100% methanol for 20 min, following with Wright-Giemsa (Beyotime, China) staining. A differential cell count of BALFs’ cell pellets was performed under a light microscope at a magnification of 200 × according to morphological characteristics.

#### Enzyme-linked immunosorbent assay (ELISA)

The trachea was dissected in the anesthetized rats, followed with pre-cooled PBS (1 ml) injection. The PBS solution was kept in the pulmonary for 1 min and then gently extracted. A total of 3 ml of the lavage fluid was collected and centrifuged for 20 min at 1000 × g, and the supernatant was harvested. The cytokines (TNF-α, IL-1β, IL-8, and IL-6) of the supernatant were detected by ELISA kits (R&D System, Minneapolis, MN, USA) according to the manufacturer's protocols. The process was repeated three times.

#### Immunohistochemistry (IHC)

The formalin-fixed, paraffin-embedded (FFPE) specimens of the lung tissue in rats and mice were cut into 5 μm sections, and deparaffinized by using xylene for 10 min twice at room temperature (RT). Heat-induced epitope retrieval was performed in 1 mM Tris buffer (1 mM EDTA, PH 9.0) for 20 min. And then the sections were incubated with primary antibody of anti‑CD68 (14-0688-82, Invitrogen, USA), diluted with 1:500 at RT for two hours. The secondary rat-anti-mouse antibody conjugated with HRP (18-4015-82, Invitrogen, USA) was added on the sections for one hour after washing the primary antibody. The protein level of CD68 was determined by staining with DAB (Diaminobenzidin) (Sigma Aldrich, Saint Louis, MO, USA) and confirmed by two pathologists (Wu and Hou) in Shanghai Pulmonary Hospital.

#### High-performance liquid chromatography (HPLC)

The molecular of RIS lacks the structure of chromophore, which we detected the concentration of RIS in lung tissue using precolumn derivatization method of HPLC [18, 19]. The amino group in RIS is used to combine with 9-fluoronylmethyl chloroformate (FMOC-Cl) to form a fluorescent group. Briefly, lung tissues (0.5 g) of rats were firstly grinded with 300 μl of standard solution. After precipitating with 200 μl of potassium dihydrogen phosphate solution (0.1 mol/L), 200 μl of calcium chloride solution (0.1 mol/L), and 400 μl of sodium hydroxide solution (1 mol/L), the precipitates were dissolved in 1 ml of sodium acetate buffer solution (0.2 mol/L, pH 4.5). The solution was then derivatized by Bond ELUT-DEA (ethylenediamine adsorption column), and ultimately RIS concentration was detected by a fluorescence detector of HPLC system (Shimadzu LCsolution, Janpan).

#### Statistical analysis

Data are expressed as mean ± SEM unless otherwise specified. One-way analysis of variance (ANOVA) using SPSS20.0 (version 20.0, SPSS, Inc., Chicago, IL, USA) software, and Graph Pad Prism 9.0 software (GraphPad, San Diego, CA, USA) was performed to compare the mean differences. A statistical difference was accepted at a *p* -value of < 0.05.

The details of immunofluorescence (IF), Hematoxylin-and-Eosin (H&E) staining, and western blot (WB) assay were documented in the Additional file 1: Methods.

## Results

### Verifying the airway obstruction of COPD rats and mice

**Fig. 1:**
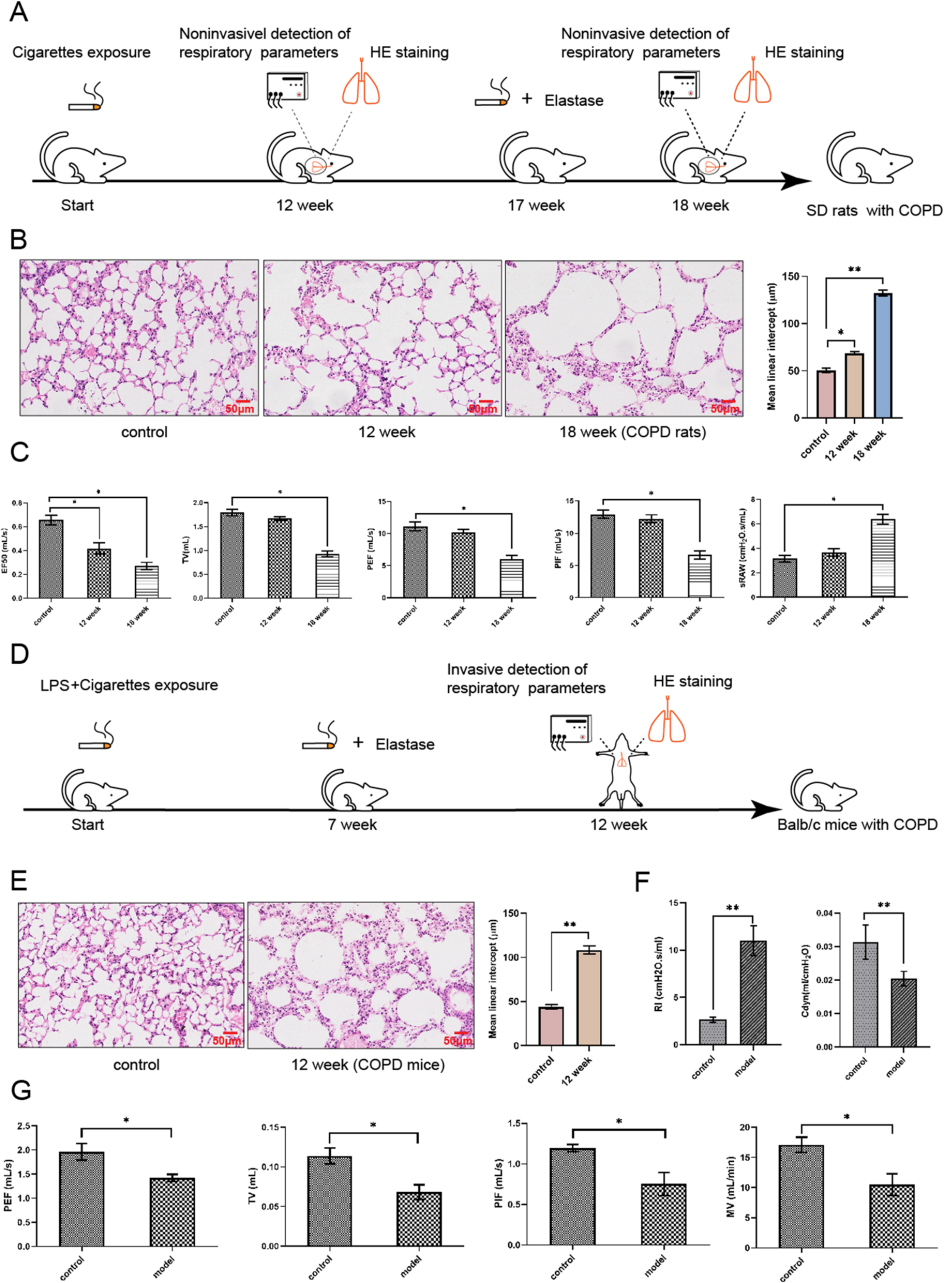
The establishment and validation of COPD in rats and mice. **A** The timeline for constructing the model of COPD in rats. **B** The HE staining of the pulmonary in COPD-rats at week 12 and 18 compared to the control group, and between-groups differences in MLI. Scale bar, 50 µM. **C** The change in respiratory parmaters, including EF50, TV, PEF, PIF, and sRAW in normal rats, rats exposed to cigarette-smoking for 12 weeks, and rats exposed to cigarette-smoking for 18 weeks and elastase injection. **D** The timeline for constructing the model of COPD in mice. **E** The HE staining of the pulmonary in COPD mice at week 12 compared with the control group, and the between-group differences in MLI. Scale bar, 50 µM. **F**, **G** The pulmonary function change, including RI/Cdyn (**F**), PEF PIF, TV, and MV (**G**), in normal and COPD mice at 12 week

The COPD rats and mice were induced through long-term exposure to cigarette smoke and a combination of elastase injection or LPS stimulation (Fig. 1A, D). The lung histology and respiratory parameters of COPD models were directly evaluated at week 12 (rats and mice) and week 18 (rats). The rapidly destroyed lung parenchymal with airspace enlargement and mean linear intercept (MLI) elevation were observed in COPD rats and mice models (Fig. 1B, E). Increased sRAW and RI representing bronchoconstriction were detected in COPD rats and mice, respectively. Decreased ventilatory parameters, EF50, Cdyn, TV, MV, PEF, and PIF were also identified in COPD animal models (Fig. 1C, F, G). The COPD symptoms of activity decrease, breath shortness, lean, and appetite loss were also observed in the animal models (data not shown).

### Risedronate inhalation ameliorated the airway obstruction in COPD rats and mice

**Fig. 2:**
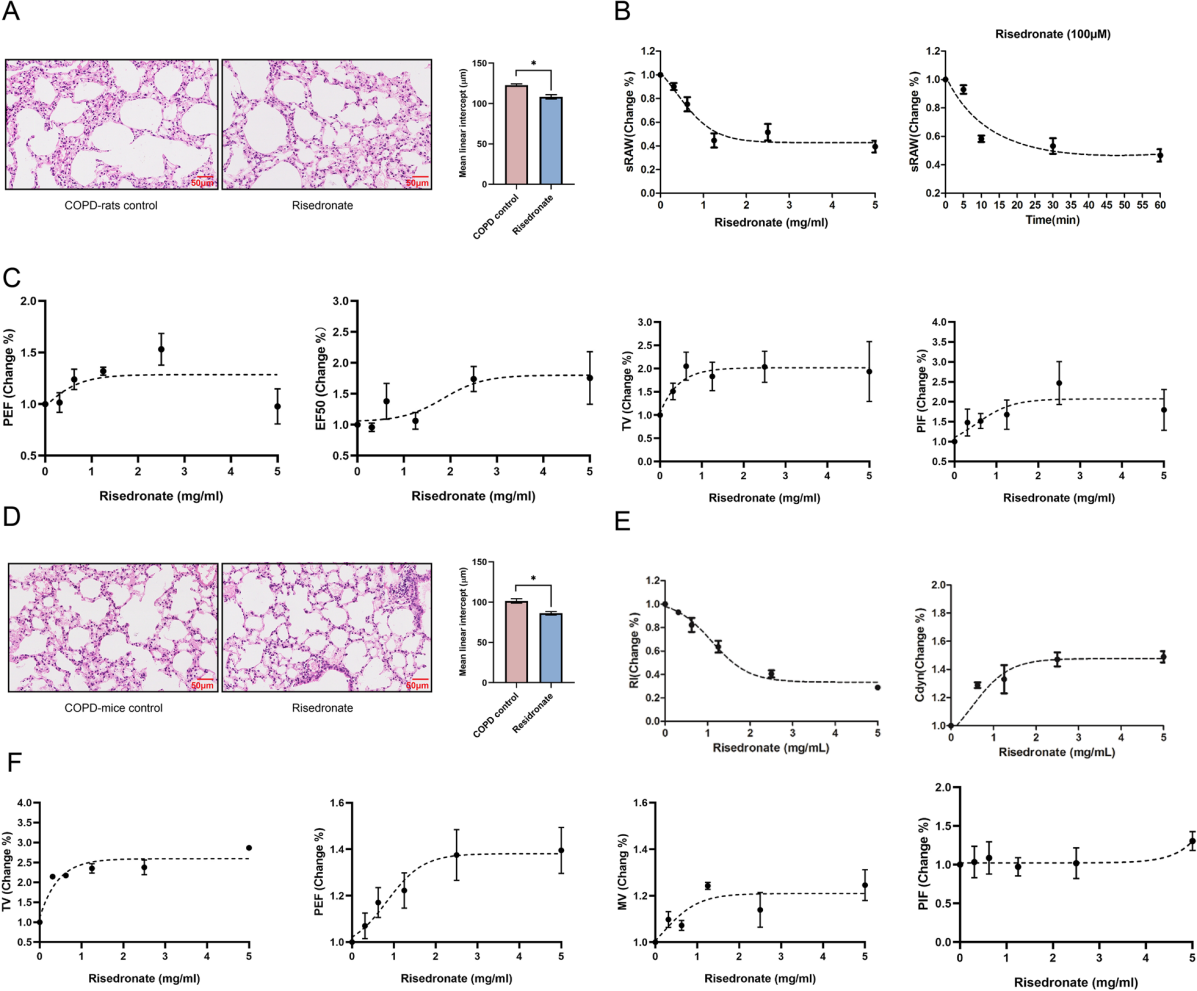
Risedronate attenuated the airway obstruction. **A** The Hematoxylin and Eosin (H&E) staining of the pulmonary section from COPD- rats (left) and those that received risedronate inhalation treatment (right). The histogram representing the differences of the two groups in the MLI. Scale bar, 50 µM. **B** The change in sRAW in COPD rats treated with different concentrations of risedronate (left) and those treated with 100 µM risedronate at different times (right). **C** The change in PEF, EF50, TV, and PIF (from left to right) in COPD rats treated with different concentrations of risedronate (0, 0.31, 0.625, 1.25, 2.5, 5 mg/ml). **D** The Hematoxylin and Eosin(H&E) staining of a pulmonary section in COPD mice (left) and those that received risedronate treatment (right). The histogram compares the two groups in the MLI. Scale bar, 50 µM.** E** The change in RI and Cdyn in COPD mice treated with different concentrations of risedronate (0, 0.31, 0.625, 1.25, 2.5, 5 mg/ml). **F** We tested the change in TV, PEF, MV, and PIF (from left to right) in COPD mice treated with different concentrations of risedronate (0,0.31, 0.625, 1.25, 2.5, 5 mg/ml)

The concentration of RIS in lung tissues was monitored as 88.8% relative to the input dose by HPLC method after inhaling RIS for five minutes, which could verify the air dispersion and bioavailability of RIS in the lung of COPD rats (Table 1). The enlarged alveolar airspace and increased MLI of lung tissues were significantly ameliorated in COPD rats and mice after RIS inhalation (Fig. 2A, D). We then detected the RIS effect on lung function by comparing the COPD animals between treated with or without RIS (%change). As the concentration and incubation time of RIS increased, the sRAW of rats decreased by approximately 60% (Fig. 2B). The other respiratory parameters, PEF, EF50, TV, and PIF increased by 15%-50% in COPD rats treated with risedronate inhalation (Fig. 2C). The coincidental results of a 60% decrease in RI and a 20–60% increase in Cdyn, TV, PEF, MV, and PIF were observed in COPD mice treated with risedronate inhalation (Fig. 2E, F).

### Risedronate inhalation attenuated the airway inflammation in COPD rats and mice

**Fig. 3:**
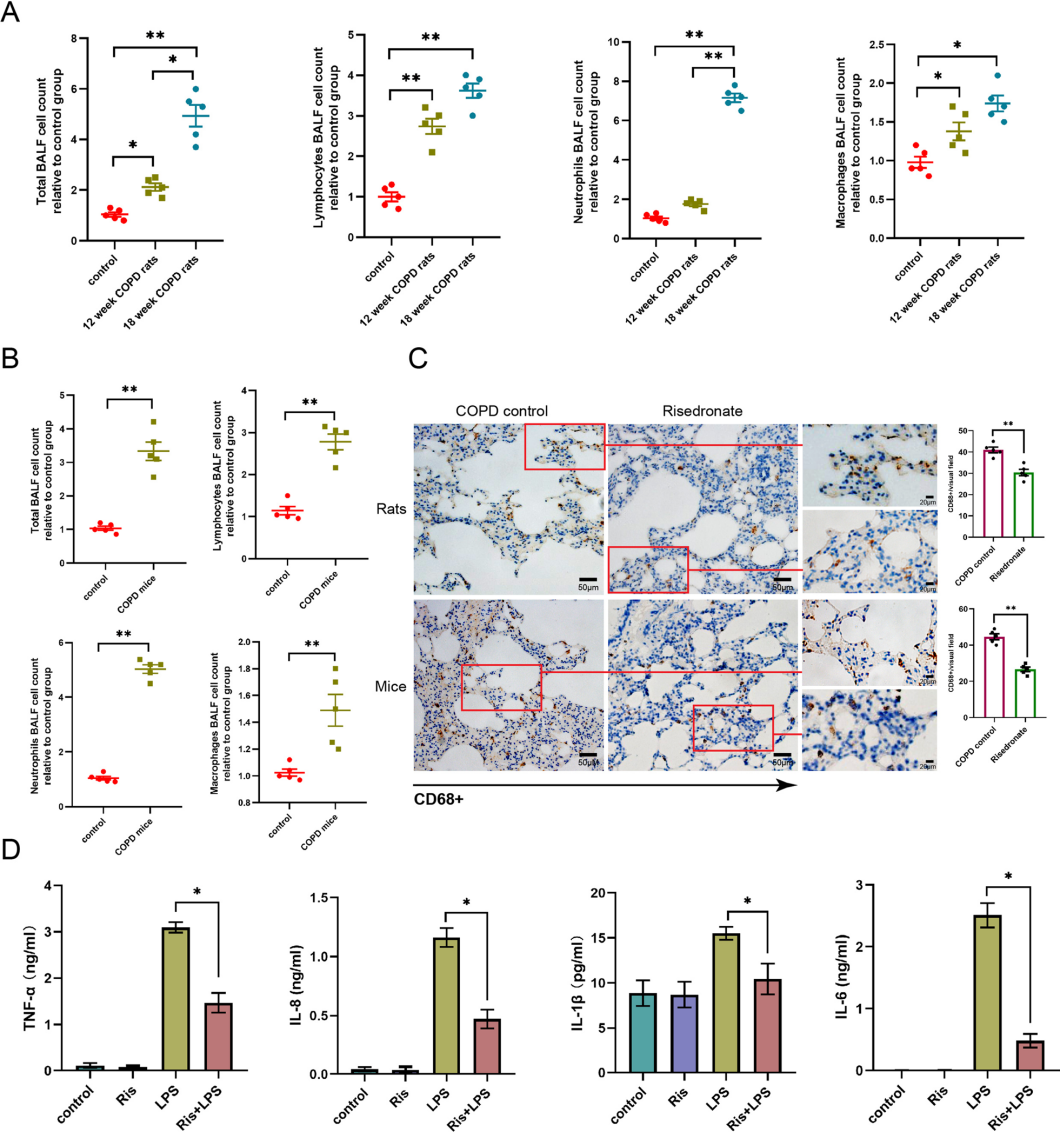
Risedronate inhibited inflammation in COPD rats and mice. **A** The cell counts of the lymphocytes, neutrophils, and macrophages in the BALF of COPD rats at week 12 and 18. **B** The cell counts of the lymphocytes, neutrophils, and macrophages in the BALF of COPD mice. **C** Detection of alveolar macrophage biomarker, CD68^+^ on the lung section of COPD rats and mice treated with or without 100 µM risedronate by immunohistochemistry staining (left: normal COPD rats or mice’ lungs; right: COPD rats or mice’ lungs treated with risedronate). **D** The change in TNF-α, IL-8, IL-1β, and IL-6 stimulated by LPS in NR8383 cells treated with 100 µM risedronate, detected by ELISA (from left to right: TNF-α, IL-8, IL-1β, and IL-6). *: *p* < 0.05; **: *p* < 0.01

Chronic lung inflammation was one critical characteristic of COPD. The increase of lymphocytes, neutrophils, and macrophages infiltration was observed in the bronchoalveolar lavage fluid of COPD rats and mice (Fig. 3A, B). In the inflammatory cells, the macrophages’ infiltration was considered the pivotal factor for emphysema[20]. We discovered the reduced macrophage biomarker (CD68 +) in lung tissues of COPD rats and mice after 2.5 mg/ml RIS inhalation, signifying the suppression of lung macrophages’ infiltration (Fig. 3C). Simultaneously, we also detected the decrease of inflammatory cytokines, TNF-α, IL-1β, IL-8, and IL-6 in the NR8383 macrophage treated with RIS, confirming the anti-inflammatory effect of RIS (Fig. 3D).

### Risedronate induced ASM relaxation by inhibiting RAS and RhoA prenylation of ASMCs

**Fig. 4:**
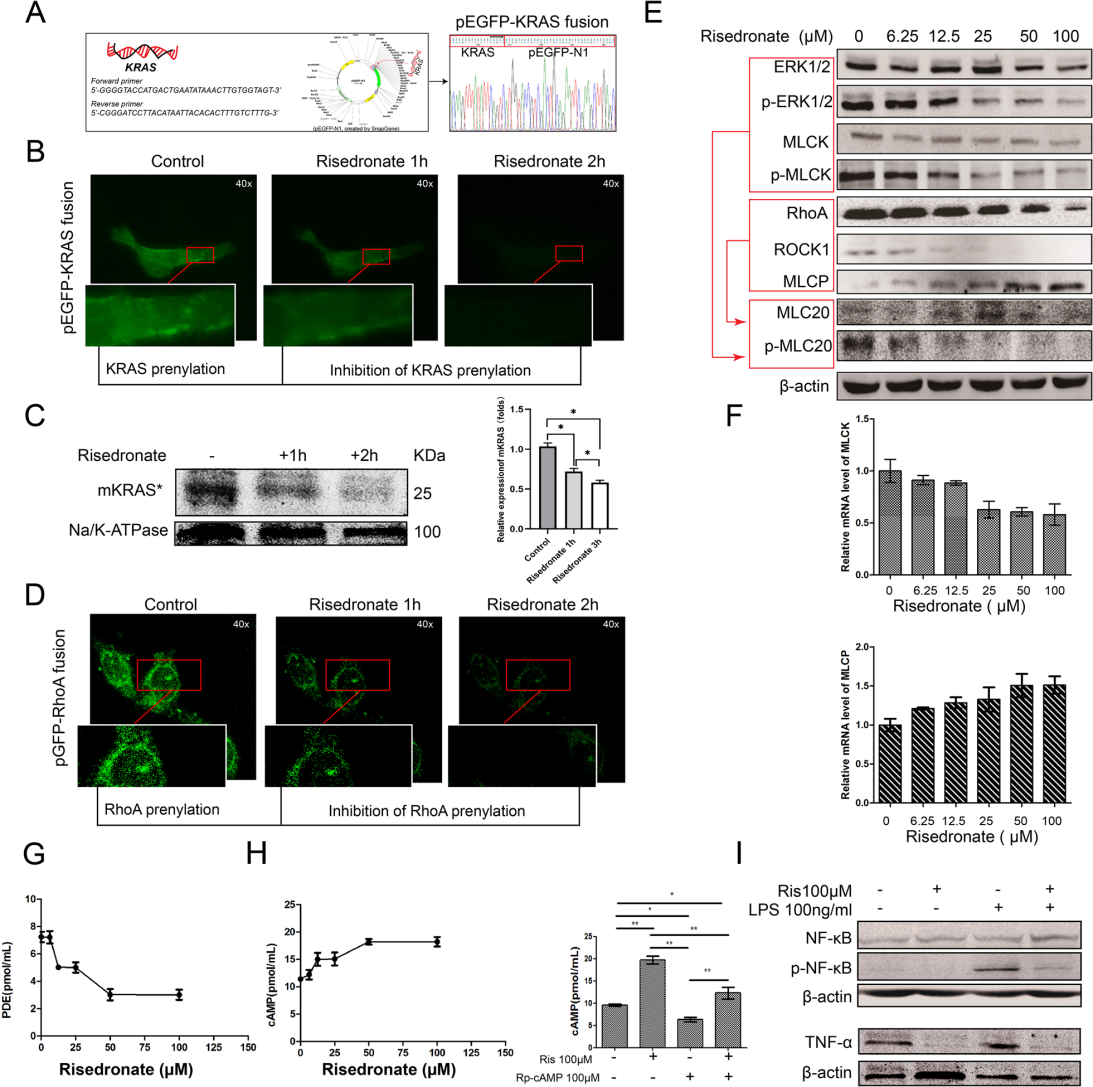
The molecular mechanism of risedronate efficacy in ameliorating the airway obstruction and inflammation response of COPD. **A** Recombination of *KRAS* into pEGFP-N1 plasmid to construct the pEGFP-KRAS fusion plasmid and validating it by sanger sequencing. **B** Transfected the pEGFP-KRAS fusion plasmid into ASMCs cells and the change in green fluorescence after incubation of risedronate for 1 h and 2 h. Scale 40x. **C** Extraction of the membrane protein and detection of the membrane KRAS in ASMC cells treated with 100 µM risedronate. **D** The pGFP-RhoA plasmid transfected into ASMCs cells and the change in green fluorescence after incubation of risedronate for 1 h and 2 h. Scale 40×. **E** The expression of ERK, p-ERK, MLCK, p-MLCK, RhoA, ROCK1, MLCP, MLC20, and p-MLC20 detected by western blot in ASMCs of rats treated with 0, 6.25, 12.5, 25, 50, 100 µM risedronate. The red frame and arrow represented RAS and RhoA-mediated two pathways of ERK-MLCK-pMLC20 and ROCK1-MLCP-MLC20, respectively. **F** The mRNA level of MLCK and MLCP in ASMCs of rats treated with 0, 6.25, 12.5, 25, 50, and 100 µM risedronate. **G** The activity of PDE in NR8383 cells treated with different concentrations of risedronate. **H** Left: Testing the activity of cAMP in NR8383 cells treated with different concentrations of risedronate. Right: cAMP inhibitor, Rp-cAMP could reverse the increase in cAMP-induced by risedronate in NR8383 cells. **I**: The expression of NF-κB, p-NF-κB, and TNF-α stimulated by 100 ng/ml LPS and its change after accepting the 100 µM risedronate incubation in NR8383 cells. *: *p* < 0.05, **: *p* < 0.01; mKRAS*, membrane KRAS; Ris, risedronate

To investigate the molecular mechanism of RIS on COPD ASM, a synthetic pEGFP-KRAS fusion plasmid and GFP-RhoA plasmid were constructed and transfected to ASMCs by lipofectamine 3000 (Fig. 4A). FDPS, one critical regulator of mevalonate pathway, could catalyze the membrane localization of GTPases, such as RAS and RhoA, through regulating the post-translational prenylation of GTPases [21]. We observed a reduced KRAS and RhoA green fluorescence and protein expression in ASMCs’ membrane after RIS incubation for 2 h (Fig. 4B–D). As an inhibitor of FDPS, RIS could decrease the membrane binding of KRAS and RhoA by inhibiting the KRAS and RhoA prenylation in ASMCs.

As the treated concentration of RIS increase, the gradual decline of KRAS downstream signaling ERK1/2, p-ERK1/2, MLCK (myosin light-chain kinase), and p-MLCK (phosphorylation of MLCK) was observed in ASMCs. The degressive of RhoA and ROCK1 (rho-associated, coiled-coil-containing protein kinase 1) and the increase of MLCP (myosin light chain phosphatase) were discovered in ASMCs treated with RIS (Fig. 4E). MLCK could phosphorylate myosin light chain 20 (p-MLC20) to trigger ASM contraction. MLCP catalyzes the dephosphorylation of p-MLC20 to induce ASM relaxation [22]. The decrease of p-MLC20 and increase of MLC20 also observed in ASMCs treated with RIS (Fig. 4E). RT-PCR also confirmed the RIS-induced decrease in MLCK and increase in MLCP, which ultimately rendered the relaxation of ASM (Fig. 4F). Therefore, the ASMCs relaxation triggered by RIS associated with prenylation-dependent RAS-ERK-MLCK-pMLC20 and RhoA-ROCK1-MLCP-MLC20 signaling pathway inhibition.

RIS could suppress lung inflammation by inhibiting the macrophages’ infiltration. We found a decrease in the PDE (phosphodiesterase) and an increase in cAMP (cyclic AMP) in NR8383 macrophage cells treated with RIS (Fig. 4G, H). A cAMP inhibitor (Rp-cAMP) in NR8383 cells treated with RIS could reverse the cAMP increase (Fig. 4H). As an essential regulator of inflammatory activities, cAMP increase reduced the production of pro-inflammatory mediators in numerous immune cells. The decline of phosphorylated NF-κB and TNF-α was discovered in NR8383 cells after RIS incubation, while the total NF-κB protein level was uninfluenced (Fig. 4I).

**Fig. 5:**
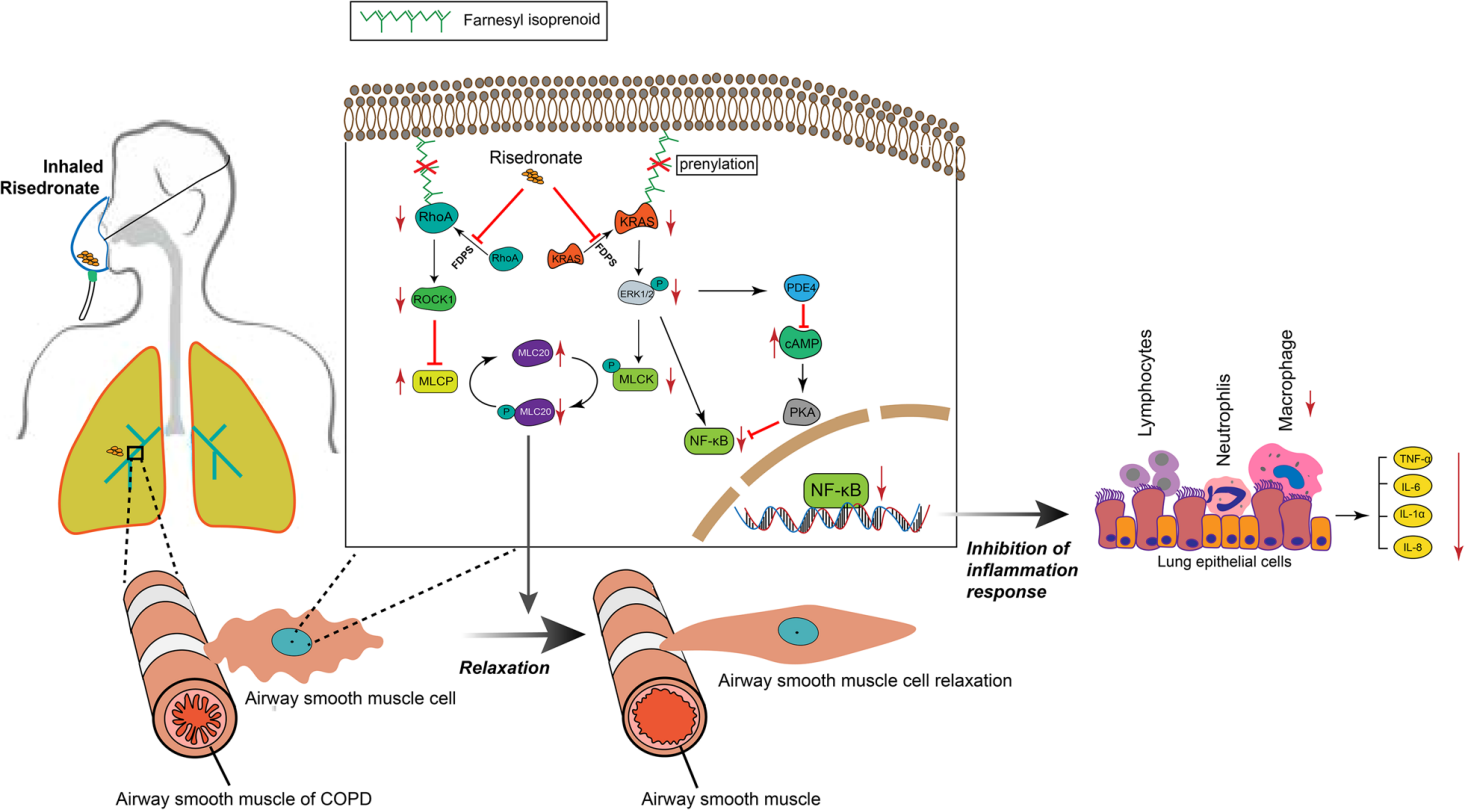
The molecular diagram of the efficiency of risedronate on COPD. Risedronate could inhibite FDPS and then disrupte the RAS and RhoA prenylation to impede its membrane binding, ultimately resulting in inactivation of RAS-ERK-MLCK-pMLC20 and RhoA-ROCK1-MLCP-MLC20 singaling pathway. Decreased pMLC20 expanded the airway by relaxating ASMCs. Decreased ERK1/2 could directely or indriectly inhibite NF-κB pathway, leading to the inhibition of inflammation response and the infiltration of lymphocytes, neutrophils and macrophages

Therefore, we confirmed the effect of RIS on ASM relaxation by inhibiting the FDPS-RAS-ERK-MLCK-pMLC20 and FDPS-RhoA-ROCK1-MLCP-MLC20 pathways. The anti-inflammatory effect of RIS involved the suppressing immune cells and macrophages' infiltration was generated through down-regulating the PDE-cAMP-NF-κB pathway (Fig. 5).

### Risedronate overcomes β2 agonist tolerance in COPD rats

**Fig. 6:**
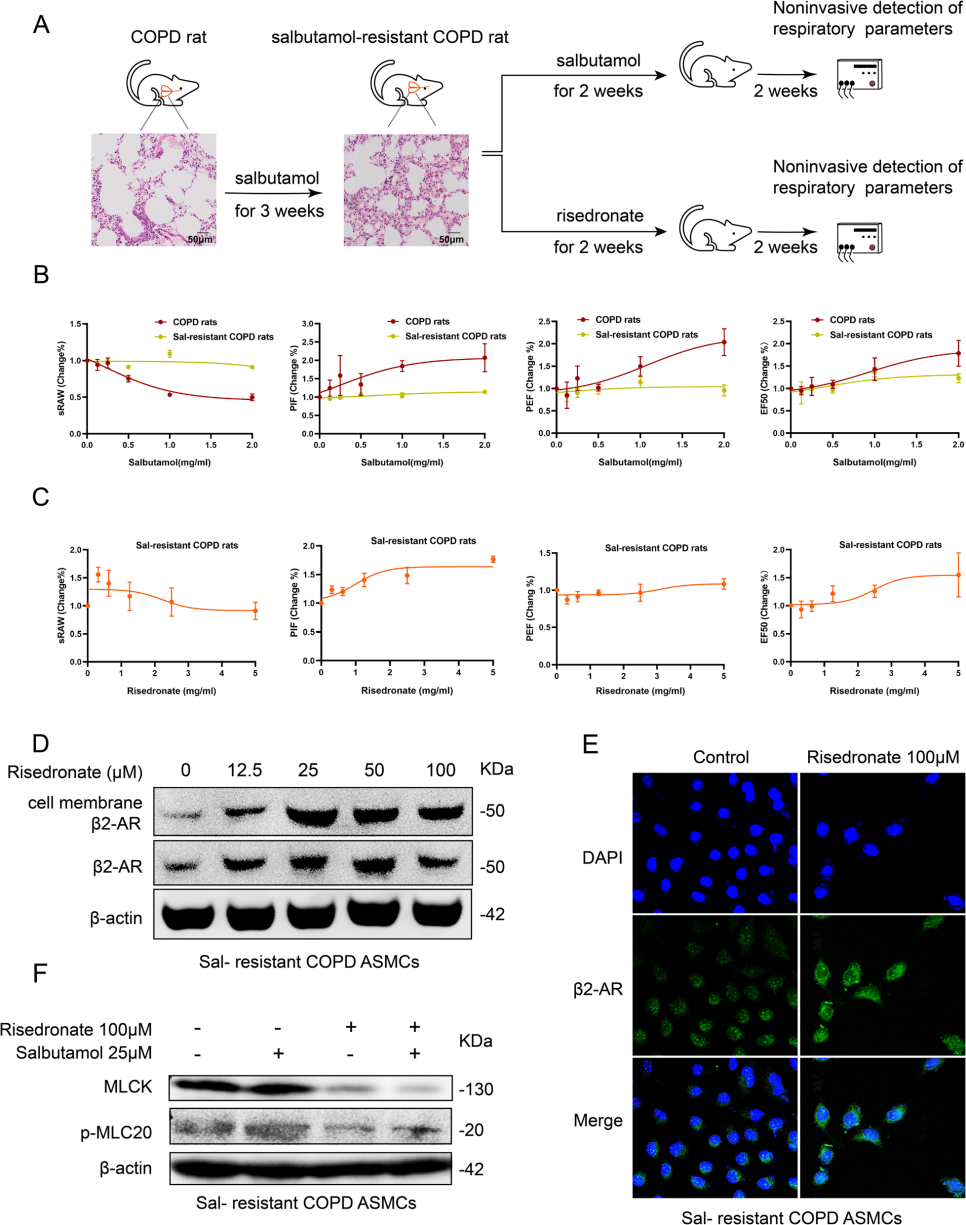
Risedronate overcomes the salbutamol tolerance of COPD rats. **A** The timeline for inducing the salbutamol tolerant COPD-rats and validating the lung structure by HE staining. **B** Validating the change in sRAW, PIF, PEF, and EF50 between COPD rats and salbutamol-tolerant COPD rats treated with different concentrations of salbutamol (salbutamol concentration: 0, 0.125, 0.25, 0.5, 1, 2 mg/ml). **C** Estimating the change in sRAW, PIF, PEF, and EF50 in salbutamol-resistant COPD rats treated with different concentrations of risedronate (risedronate concentration: 0, 0.31, 0.625, 1.25, 2.5, 5 mg/ml). **D** Detection of β2-AR and the membrane β2-AR in ASMCs of salbutamol-tolerant COPD- rats treated with different concentrations of risedronate by WB assay (risedronate concentration: 0, 12.5, 25, 50, 100 µM). **E** Detection of β2-AR and the membrane β2-AR in ASMCs of salbutamol-tolerant COPD- ratstreated with 100 µM risedronate by IF assay. **F** Detection of the MLCK and p-MLC20 in ASMCs of salbutamol-resistant COPD rats treated with 25 µM salbutamol and/ or 100 µM risedronate by WB assay. *Sal-resistant,* salbutamol-resistant

The inhaled β2 agonists are commonly used to reduce the symptom of air-flow obstruction in COPD patients, although their long-term use could increase tolerance. We constructed the β2 agonist (salbutamol)-tolerant COPD rats to explore the effect of RIS. When treated with salbutamol, the β2 agonist (salbutamol)-tolerant COPD rats had higher sRAW and lower PIF, PEF, and EF50 than COPD rats (Fig. 6A, B). After RIS inhalation, the pulmonary function of salbutamol-tolerant COPD rats was partly rescued by sRAW decline and PIF, PEF, and EF50 elevation (Fig. 6C). The down-regulation of β2-AR was correlated with the tolerance of β2 agonists [23]. We found the increase of cell membrane β2-AR in ASMCs treated with RIS by WB and IF assay (Fig. 6D, E). Risedronate could also induce the ASMCs’ relaxation in salbutamol tolerant COPD rats through suppressing MLCK and p-MLC20 expression (Fig. 6F). Therefore, RIS might attenuate of airway obstruction in salbutamol-tolerant COPD rats through upregulation β2-AR.

## Discussion

Airway obstruction and chronic inflammation are the main characteristics of COPD [24]. For the first time, this study documented the effectiveness of RIS on both attenuating airway obstruction and inflammation. As an inhibitor of FDPS, the effect of RIS was relevant to the function of FDPS, an important regulator of mevalonate pathway, which is involved in the biosynthesis of isoprenoid intermediate, farnesyl pyrophosphate (FPP). The isoprenylation is the critical post-translational modification of the small GTP-binding proteins, such as RAS or RhoA, primarily mediating its membrane localization and downstream signaling activation [25]. Hence, inhibition of FDPS could interrupt the synthesis of FPP and Ras/ RhoA isoprenylation, resulting in the inactivation of downstream signaling. In our study, the decline of cell membrane RAS and RhoA was discovered in ASMCs treated with RIS. And the downstream RAS-ERK1/2-MLCK-pMLC20 and RhoA-ROCK1-MLCP-MLC20 signal pathways were inhibited by RIS, ultimately leading to ASM relaxation. MLCK has been identified as an ERK1/2 substrate which involved in the cell motility [26, 27]. ROCK1, a substrate of RhoA, could suppress MLCP [28–33]. MLCK and MLCP could phosphorylate MLC20 and dephosphorylate p-MLC20, ultimately rendering the contraction and relaxation of ASM, respectively [34]. The decrease of MLCK and increase of MLCP was observed in ASMCs treated with RIS, which have provided the molecular foundation of the bronchodilation in COPD rats and mice. The plethysmography method used in COPD rats and direct measuring of the airway response in COPD mice both confirmed the effect of RIS of  curbing COPD-pulmonary function decline.

The chronic inflammation of lung is central for the development of COPD. The infiltration of macrophages, lymphocytes, and neutrophils, thickening of fibrosis and small airway wall were the primary components of COPD [35]. The inhaled corticosteroids (ICS) were remarkably effective in controlling the inflammation of COPD. However, the long-term use could cause systemic side effects. Hence, it is pivotal to find an efficient and safe way for suppressing the inflammatory response of COPD. The suppression of inflammatory cells’ accumulation, especially macrophages’ infiltration was observed in COPD rats treated with RIS. The macrophages’ apoptosis induced by bisphosphonates was reported as one mechanism of ameliorating emphysema [12, 36]. In our study, the bronchodilatation was monitored immediately and obviously in COPD animals after RIS administration, and the detection time was relatively short. We then focused on the signaling pathways triggered by RIS, including the inflammatory pathway, which we found the suppression of PDE-cAMP-NF-κB pathway. And the decrease of cytokines was also detected in NR8383 macrophage after RIS treatment, which might be the consequence of reduced macrophages’ infiltration. The effect of RIS nebulization on inhibition of macrophages’ function or the apoptosis in COPD animals might need further investigation.

The abnormal enlargement of airspace and fibrosis of alveoli are the primary pathological features of COPD, and the medications could not reverse the damaged structure of the alveolar. Risedronate played the most important role in attenuating emphysema of COPD and partly reversing the remodeling airway. We observed the decrease in lung MLI and inhibition of airspace enlargement in the COPD model treated with risedronate, which might be associated with the inhibition of MMP-13 (data not show), a critical driver of airway destruction in COPD [37]. However, the 10-day treatment with risedronate might incompletely change the alveolar structure. Further studies are needed to evaluate the long-term effect of RIS on reversing damage to alveoli’s structure and fibrosis.

COPD patients treated with inhaled steroids had the high risk of osteoporosis, which was diagnosed in more than one-third of COPD patients [38, 39]. How osteoporosis impact COPD progress is unknown. Risedronate, an FDA-approved drug used for osteoporosis, the efficiency on COPD treatment provided a potential linkage between COPD and osteoporosis. Since the osteoclast developed from monocyte/macrophage lineage [40], we suspected that the inhibition of inflammation and macrophages’ infiltration by risedronate might be the bridge of COPD and osteoporosis. We might provide the probability that RIS could be used for treating COPD patients with comorbidity of osteoporosis.

Beta-2 agonists are other crucial medications used for COPD patients. However, the long-term use of β2 agonists resulted in tolerance. A previous study has reported that alendronate expands the airway by mediating the expression of β2 receptors [11]. We determined that RIS could also enhance lung function in β2-agonists tolerant COPD rats by increasing the expression of β2 receptors on the cell membrane. Risedronate is significantly important for COPD treatment. The constructed COPD animal model was limited to stable COPD conditions and not suitable for acute exacerbations of COPD.

## Conclusions

In summary, nebulization risedronate can effectively attenuate airway obstruction in COPD by relaxing the airway smooth muscles, inhibiting the inflammation response and changing the alveolar structure. RIS might be a novel reagent for COPD patients, especially those with osteoporosis complication or those with β2-agonists tolerance.

**Table 1:** Absorption of risedronate aerosol in lung tissues of rats

Rats	Lung tissues	Weight (g)	Volume (ml)	Ris con (μg/ml)	Total inhaled (mg)	Absorbed (%)
	1	0.193	0.5	51.6	0.026	8.0
	2	0.260	0.5	130.5	0.065	20.0
No.1	3	0.318	0.5	169.2	0.085	26.2
	4	0.280	0.5	124.8	0.062	19.1
	5	0.211	0.5	65.4	0.033	10.2
	Total	1.262			0.271	83.50
	1	0.242	0.5	99.1	0.050	15.4
	2	0.264	0.5	118.5	0.059	18.2
No.2	3	0.302	0.5	139.2	0.070	21.5
	4	0.356	0.5	144.2	0.072	22.2
	5	0.265	0.5	107.3	0.054	16.6
	Total	1.429			0.305	93.9
	1	0.186	0.5	75.4	0.038	11.7
	2	0.262	0.5	124.5	0.062	19.1
Mean	3	0.310	0.5	154.2	0.078	23.9
	4	0.318	0.5	134.5	0.066	20.7
	5	0.238	0.5	86.4	0.044	13.4
	Total	1.346			0.288	88.8

*Ris con* risedronate concentration

## Supplementary Information

**Additional file 1.** Methods. **Fig. S1.**The Non-Invasive Airway Mechanics Using the Plethysmography system for detecting the respiratory parameters.

## Data Availability

The datasets used and/or analyzed during the current study are available from the corresponding author on reasonable request.
