# Supplementary material for: Nebulization of risedronate alleviates airway obstruction and inflammation of chronic obstructive pulmonary diseases via suppressing prenylation-dependent RAS/ERK/NF-κB and RhoA/ROCK1/MLCP signaling
Source: Respir Res. 2022 Dec 28;23:380. doi: 10.1186/s12931-022-02274-5 (PMC9795678; doi:10.1186/s12931-022-02274-5)
Supplement: Supplementary file 1 — Additional file 1. Methods. Fig. S1.The Non-Invasive Airway Mechanics Using the Plethysmography system for detecting the respiratory parameters. [file 12931_2022_2274_MOESM1_ESM.docx]

**Additional file 1: Methods**

**Immunofluorescence**

The pEGFP-Ras fusion plasmid was constructed according to previously study [1] and validated by sanger sequence. The pcDNA3-EGFP-RhoA-wt (pGFP-RhoA) was purchased from Addgene (Addgene, #12965). The 10ng pEGFP-Ras and pGFP-RhoA plasmids were transfected to HEK293T cells with lipofectamine 3000 when cells achieved 60% confluence. After 48 hours of transfection, the liposome solutions were replaced with fresh DMEM medium containing 10% FBS, following with incubation of risedronate for additional 1 and 2 hours. HEK293T cells were digested into single cell, and 1000 cells were seeded onto a sterile cover slide in a 6-well plate with complete medium. When achieved 70% confluence, the cells were treated with 100µm RIS for 1h and 2h. And then the cells were fixed with 4% paraformaldehyde for 20 min, following with washing twice by 1x phosphate-buffered saline (PBS) and incubating with 0.2% Triton X-100 for 5 min at room temperature (RT). Five percent sterile bovine serum albumin (BSA) was utilized for blocking the antigen with 30 min at RT. The anti-KRAS (12063, Proteintech) and anti-RhoA (10749, Proteintech) first antibody was diluted with 1:100 in 1% BSA and added to the cells for overnight incubation at 4 ℃. The donkey anti-rabbit fluro 488 secondary antibodies (Ab150073, Abcam) was incubated (1:500 dilution with 1% BSA) for 45 min after removing the first antibody in cells at RT. The coverslips were mounted on slides with a fluorescent anti-quenching agent to seal the dye, and then the coverslips were observed under an Olympus fluorescence microscope (excitation 495 nm).

**Hematoxylin-and-Eosin (H&E) staining**

Hematoxylin-and-Eosin (H&E) staining was performed on paraffin - embedded lung tissue sections (4μm). The sections were dewaxed with xylene, following with gradient ethanol hydration and tap water rinsing. The hydrated sections were immersed in hematoxylin staining solution for 5-20 min to stain the nuclei, following with tap water washing for 3-5 min. The sections were then differentiated with 1% hydrochloric acid and ethanol for 5~30s, following with tap water washing 1-3 min. Using the weak alkaline aqueous solution returns to blue for 30s~1min, following with tap water washing for 5-10 minutes. The fully hydrated sections are directly put into the eosin staining solution, and the cytoplasm is stained for about 5-15 minutes. At last, the sections are dehydrated with gradient ethanol and transparent with xylene, following with neutral gum sealant. The lung tissue morphology was determined through light microscopy at a magnification of 200x.

**Western blot**

Cells was collected and lysed with radioimmunoprecipitation buffer (RIPA) buffer (Cat# 89900, Thermo Fisher) containing protease and phosphatase inhibitor cocktail (Cat# 78442, Thermo Fisher). The protein samples were isolated by sodium dodecyl sulfate-polyacrylamide gel electrophoresis (SDS-PAGE) on a 10% separating gel and electro-transferred onto a polyvinylidene fluoride (PDVF) membrane (Bio-R). The membrane with protein blots was blocked with blocking buffer (Cat# 927-40000, LICOR, US) for 1 h at RT. The anti-KRAS primary antibody (#12063, Proteintech), anti-RhoA (#10749, Proteintech), anti-Na/K-ATPase (#3010, Cell Signaling ), anti-β-actin (ab115777, Abcam), anti-ERK1/2 (#9102, Cell Signaling), anti-pERK1/2(#4376, Cell Signaling), anti-MLCK (ab232949, Abcam), anti-pMLCK (ab200809, Abcam), anti-RhoA (ab187027, Abcam), anti-ROCK1 (ab134181, Abcam), anti-MLCP (ab70809, Abcam), anti-MLC20 (M4401, SigmaAldrich), anti-pMLC20 (AB3381, SigmaAldrich), anti-NF-κB (ab16502, Abcam), anti- pNF-κB (#3031, Cell Signaling), anti-TNF-α (#3707, Cell Signaling), and anti-β2-AR (ab182136, Abcam, clone: EPR707(N)), were used and diluted with 1:1000 in Odessy blocking buffer. Blots were incubated with the first antibody overnight at 4 ºC. The antigen-antibody complex was detected with IRDye® 800CW anti-mouse (LI-COR) or rabbit (LI-COR) secondary antibody for 1 h at RT and visualized using the Odyssey® Sa Infrared Imaging System (LI-COR Biosciences).

1. Choy E, Philips M: **Green fluorescent protein-tagged Ras proteins for intracellular localization.** In *Methods in Enzymology.* *Volume* 332: Elsevier; 2001: 50-64

**Additional file 1: Figure S1: The Non-Invasive Airway Mechanics Using the Plethysmography system for detecting the respiratory parameters.**

**
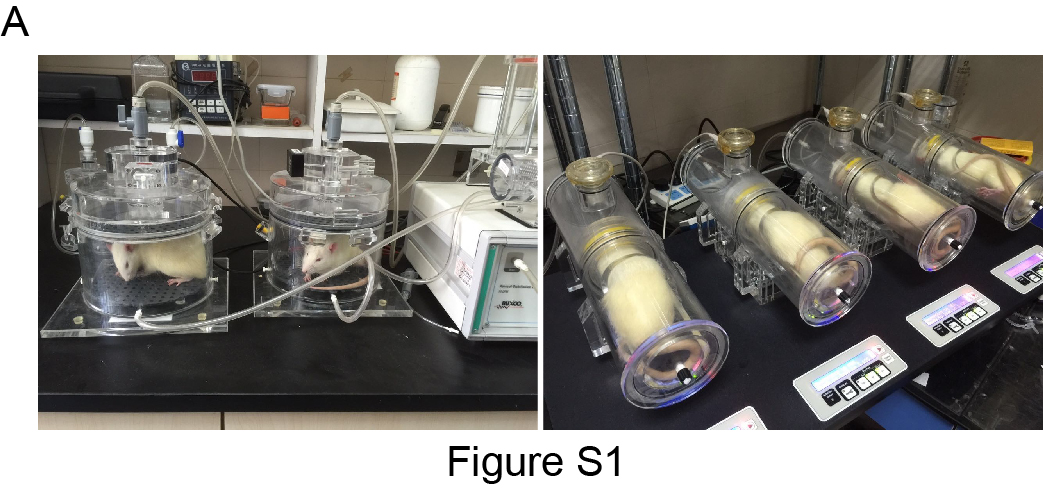
**
